# Effects on walking performance and lower body strength by short message service guided training after stroke or transient ischemic attack (The STROKEWALK Study): a randomized controlled trial

**DOI:** 10.1177/0269215520954346

**Published:** 2020-09-18

**Authors:** Birgit Vahlberg, Erik Lundström, Staffan Eriksson, Ulf Holmbäck, Tommy Cederholm

**Affiliations:** 1 Department of Public Health and Caring Sciences, Clinical Nutrition and Metabolism, Uppsala University, Uppsala, Sweden; 2 Department of Neuroscience, Neurology, Uppsala University, Uppsala, Sweden; 3 Department of Neuroscience, Physiotherapy, Uppsala University, Uppsala, Sweden; 4 Centre for Clinical Research, Sörmland, Uppsala University, Eskilstuna, Sweden; 5 Department of Community Medicine and Rehabilitation, Physiotherapy, Umeå University, Sweden

**Keywords:** Stroke, TIA, physical activity, secondary prevention, rehabilitation interventions

## Abstract

**Objective:**

To evaluate whetherdaily mobile-phone delivered messages with training instructions during three months increase physical activity and overall mobility in patients soon after stroke or transient ischemic attack.

**Design:**

Randomised controlled trial with intention-to-treat analyses.

**Setting:**

University hospital. Data collection from November 2016 until December2018.

**Subjects:**

Seventy-nine patients (mean (SD) age 63.9 (10.4) years, 29 were women) were allocated to either intervention (*n* = 40) or control group (*n* = 39). Participants had to be independent (modified Ranking Scale ⩽2) and able to perform the six-minute walking test at discharge from the hospital.

**Interventions:**

The intervention group received standard care and daily mobile phone instructional text messages to perform regular outdoor walking and functional leg exercises. The control group received standard care; that is, primary care follow-up.

**Main measures:**

Walking performance by six-minute walking test (m), lower body strength by five times chair-stand test (s), the short physical performance battery (0–12 points) and 10-metres walk test (m/s) were assessed at baseline and after three months.

**Results:**

The estimated median difference in the six-minute walking test was in favour of the intervention group by 30 metres (95% CI, 55 to 1; effect size 0.64; *P* = 0.037) and in the chair-stand test by 0.88 seconds (95% CI, 0.02 to 1.72; effect size 0.64; *P* = 0.034). There were no differences between groups on the short physical performance battery or in 10-metres walking time.

**Conclusions:**

Three months of daily mobile phone text messages with guided training instructions improved composite mobility measures; that is, walking performanceand lower body strength.

**Clinical Trial Registry:**

The study is registered with ClinicalTrials.gov, number NCT02902367.

## Introduction

Physical inactivity after stroke increases the risk of cardiovascular disorders and is associated with decreased mobility and functional independence.^1–3^ Therefore, physical activity early after a stroke as part of secondary prevention is emphasised in several clinical guidelines.^3,4^ Thigh muscle strength is important for the ability to stand up from sitting, climbing stairs, and for walkingperformance. In addition, muscle fitness is important for glucose metabolism and cardio-respiratory vigour.^3,5^

Stroke survivors as well as individuals with transient ischemic attacks usually display continuous cardiovascular risk, including low physical activity and time spent in sedentary behaviours.^6^ An observational study indicated that following a stroke, people spend close to 11 hours sitting per day, mostly in prolonged bouts.^2^ Community-living individuals after a stroke are 23% less physically active compared to individuals at the same age without stroke.^7^

It has been observed that attempts to increase physical activity after stroke are not always successful.^4^ This could partly be due to a lack of models for supervised training adapted for individuals post stroke or transient ischemic attack.^8,9^ Thus, it is vital to find user-friendly rehabilitation alternatives in order to increase physical activity.^5^ Current Swedish guidelines promote early non-pharmacological secondary prevention efforts after all types of cerebrovascular events including transient ischemic attack, but details about effective methods of delivery are lacking.^3,10^ For example, current recommendations do not include the use of pedometersor training diaries.

To date, the use of mobile phones to facilitate training for persons soon after mild stroke or transient ischemic attack needs further exploration. The aim of the STROKEWALK Study was to evaluate a three-month programme consisting of daily mobile phone instructional text messaging (short message service/SMS), combined with a two-week use of a pedometer to record walking distance, and the use of a training diary. The hypothesis was that this programme would be better than the current standard treatment for affecting overall mobility, including walking performance and lower body strength.

## Methods

### Design and participants

The STROKEWALK Study was a single-centre, parallel-group, randomised controlled non-blinded clinical trial performed at the stroke-unit at the Uppsala University Hospital, Sweden. The study was performed according to the Helsinki declaration andethical approval was obtained from the regional Ethical Review Board of Uppsala University Hospital, Sweden: Dnr: 2015/550. The study was registered with ClinicalTrials.gov (NCT 02902367). All participants provided written informed consent. Full details about the study protocol (design, recruitment, and intervention) have been described in detail elsewhere.^11^ Recruitment started November 1 2016, and ended September 18 2018, when the target number of participants was reached.Thelast three-month follow-up examination was performed December 19 2018. The present study was supported by grants from the Medical faculty at Uppsala University, Swedish Stroke Association (STROKE-Riksförbundet), the Uppsala County Council and the Swedish Association of Physiotherapists, Neurology.

Potential participants were identified by regular screening of the patient lists at the stroke-unit. Inclusion criteria were a verified acute stroke (infarction or intracerebral haemorrhage) or transient ischemic attack as first or recurrent event; age ⩾18 years; planned discharge to independent living; and having a mobile phone. Other inclusion criteria were absence of cognitive impairment, that is, Montreal Cognitive Assessmentscale ⩾23 points,^12^ good motor function definedasa modified Rankin Scale score of ⩽2,^13^ and sufficient walking ability to perform the six-minute walking test with or without walking aid at the time of discharge from the hospital.^14^ Patients were excluded if they had known subarachnoid haemorrhage, medical problems such as uncontrolled hypertension, untreated arrhythmias, unstable cardiovascular conditions, a dementia diagnosis, severe aphasia, severe psychiatric problems or cognitive impairment with difficultiesto understand instructions.

The allocation procedure for the experimental or the control group was performed by a research-assistant within one week after the baseline measurements. The allocation scheme was not revealed to the physiotherapist who enrolled the patients. The group allocation was based on simple randomisation and a computerised random distribution stratified by gender, pre-arranged and performed with closed envelopes. Block allocation was not used. One series of envelopes for men and another for women were used. The envelopes for allocation were kept in a locked room.

### Assessments

All baseline data were collected at one occasion while the participants were still being treated at the hospital or soon after discharge from the hospital. One experienced physiotherapist (BV) performed the assessments. Assessments of overall mobility as described below were performed at baseline and after three months at the hospital.

The general baseline evaluation and assessment included:

- Smoking habits and education level, assessed by yes or no answers to the questions: ‘Are you a smoker at this time of your life?’ and ‘Do you have a university degree?’
- Cardio-metabolic risk factors and diagnoses such as diabetes, hypertension, hypercholesterolemia and cardiac heart failure were registered from the patient’s medical records. Supine blood pressure was measured manually, and the last registration before discharge from the hospital was recorded.
- Co-morbidity was assessed according to the Charlson Comorbidity Index using information on diagnosis from the medical records.^15^ The Charlson Comorbidity Index is validated to predict 10-year survival.^15^ Each current condition was given a weight based on severity and was used for the summary score. The current stroke event was not included in the summary score.
- Body mass index was calculated as the body weight (kg) divided by height (m) squared. Weight was recorded with participants wearing light indoor clothing. Height was measured to the nearest cm. Weight and height were registered by a research assistant, except for seven patients where weight at admission was retrieved from the medical records.
- The Montreal Cognitive Assessment scale (0–30 points) was used to evaluate cognitive function,^12^ a higher value indicating better function.
- The stress profile was assessed by a four-level self-reported everyday life stress scale, It combines 20 claims with agreements expressed in terms of time urgency/impatience or easily aroused irritation/hostility; for example, ‘Other people’s mistakes irritate me’.^16^ This questionnaire refers to stress behaviours in everyday life situations. It scores from not at all (0) to fully agree (3).
- The modified Rankin Scale was used to assess motor function and global disability.^13^ It is scored from 0 (no symptoms) to 6 (dead), with 2 indicating slight disability, unable to perform all previous activities, but able to look after one’s own economy without assistance.
- The four-level Saltin-Grimby Physical Activity Level Scale was used for interviews to register self-reported physical activity the year before the stroke^17^; that is, physical inactivity (sedentary), some light physical activity, regular moderate physical activity and hard physical training for competitive sports.

### Primary and secondary outcomes

The study included two primary outcomes: the six-minute walking test (metres) to reflect walking performance,^14^ and the chair-stand test (seconds) to reflect lower body strength, including thigh muscle strength.^18,19^ The six-minute walking test measures the maximal walking distance during six minutes over a 30 mcourse.^14^ For the chair-stand test the participant is instructed to rise up from a seated position without support as quickly as possible for five times in a row.^18^ The tests were performed with standardised instructions. The chair-stand test is a subscale of the short physical performance battery. In the original study protocol, the chair-stand test was not designed to be a co-primary outcome, and not mentioned as such in the trial registration. This change was considered early after trial registration, and is described in the pilot study.^11^

Secondary outcomes were the 10-metre walk test (m/s) and the Short Physical Performance Battery (0–12 points). The 10-metre walk test measures comfortable walking speed registered with a stopwatch.^20^ It gives an indication of the gait function in people with neurological impairments.^21^ The short physical performance battery^18^ includes assessments of balance (scores), gait speed (3 metres, m/s) and the chair stand test (seconds). In that test, balance was assessed as the ability to stand unsupported for 10 seconds or more, with feet together or in semi tandem (heel of one foot placed by the big toe of the other foot)or in full tandem (feet directly in front of each other). Gait speed was measured with a three metre long distance and the individuals were instructed to walk in their comfortable speed.Each item is graded from zero (unable to perform the test) to four points, providing a total score of 0–12 points, with a higher score indicating better mobility. In the trial registration, it is indicated that the study also will provide data on body composition, cardio-metabolic risk-factors, grip-strength, food intake and self-reported health. These data are under analyses and will be presented separately.

### The intervention

The intervention consisted of three components: (1) daily mobile-phone delivered instructional text messages via short message services (i.e. SMS) for three months, (2) the use of training diaries for three months and (3) pedometers for the registration of step counts during week 1 and week 12; that is, beginning and end of study.The text messages gave instructions on how to exercise to increase walking performance and improve lower body strength and were delivered through an internet service (www.intime.nu).^11^ The communication was only in one direction; that is, the participants of the intervention group received the training instructions without being able to text back to seek help or advice.

The training programme was initiated about one week after the patients had been examined and randomised. Participants were asked to walk ten minutes daily at a moderate intensity (12–13 on the Borg exertion scale, see below) for the first two weeks, and then gradually increase the walking time and the exertion to a perceived strenuous intensity by the third month up to 15 on the Borg exertion scale.^22^ The Borg scale measures self-monitored perceived rate of exertion that ranges from 6 (nothing at all) to 20 (maximal). During the third month of the intervention, the outdoor walking was performed either as a 30-minute walk or in intervals.

Moreover, a functional lowerbody exercise was described in the daily text messages; that is, to repeatedly rise from a sitting position without support. The number of repetitions were gradually increased, starting from 10 rises to 15 rises repeated three times per day. Patients were instructed to rest one day per week, that is, being active but not necessarily doing walking exercises. Some examples of text messages in Swedish and in English are provided in the Appendix 1. The cost for sending text messages was borne by the research programme and amounted per individual to 50 Euros per three months (as a subscription fee) and an additional cost of five cents per text message.

Adherence with the training programme was assessed in the intervention group by a training diary that included the Borg scale assessments. The participants registered the exercise they had performed, the duration, and intensity on a daily basis through the intervention period.

The participants in the intervention group were also equipped with a pedometer that registered the number of stepsdaily during the first and the last week of the three months intervention. This was considered sufficient to calculate a change in number of steps throughout the three months intervention. The pedometer/moving sensor Yamax LS20000 (Yamax Corporation, Tokyo, Japan) was used. Participants were instructed to wear the pedometer at all times during the day, except for when sleeping, bathing, or swimming. According to Tudor Locke^23^ walking <5000 steps/day is defined as having a ‘sedentary lifestyle index’, 5000–7499 steps/day is considered ‘low activity’, 7500–9999 steps/day is considered ‘somewhat active’, approximately 10,000 steps/day is defined as ‘being active’, and >12,500 steps/day is classified as ‘highly active’.

### Control group

Patients in the control group were given standard stroke unit care, which usually does not include specific advice about physical activity or home exercise programmes including walking and sit to stand practice. They were given standard recommendations, with no restrictions regarding physical activity, exercise or taking part in rehabilitation services.

In general, following a stroke or transient ischemic attack, patients are referred to their general practitioner for a meeting within three months after discharge for further risk factor management. Some patients with special needs receive immediate rehabilitation or follow-up after discharge from the stroke-unit. This is provided by the hospital at an out-patient clinic or in the patient’s home.

### Statistics

Power calculations indicated that 80 individuals (including an anticipated dropout rate of 20%) were required for 80% power with alpha = 0.05 to detect a 34-metre clinically relevant mean difference in the six-minute walk test with a standard deviation of ±43 m.^24^ The assumptions were based on variations previously reported on the effects of a progressive resistance exercise programme in post-stroke individuals as well as earlier reported difference in changes in the six-minute walking test.^24–26^ A conservative intention-to-treat analysis was applied for differences in changes between the intervention and control groups, that is, for all missing values (dropouts), the change was assumed to be zero. Thus, follow-up data for dropouts were registered with a carry-forward approach. Descriptive data are reported as mean (SD) and median (IQR). To check for normal distribution, the Shapiro-Wilk W test and histogram viewing were used.

Differences in changes between baseline and the three-month follow-up between the intervention and the control groups are presented with 95% CI and tested by non-parametric methods (Mann-Whitney U test) for ordinal or non-normally distributed variables. The median differences in changes were calculated as Hodges-Lehmann estimates. The effect size of the primary outcome measures and between-group differences was calculated using the Hodges-Lehmann estimator^27^ *and* the area under the receiver-operating characteristic curve.^28^ In addition, sensitivity analyses were performed using only complete cases.

In order to control for confounding effects, post hoc univariate and multiple logistic regression models were used to calculate the Odds Ratios (ORs) and 95%CI for higher than median versus lower than median change in the six-minute walk test as the dependent variable. The cut-off value for high versus low change was the median change of 32 metres. Similar, ORs were calculated for improved chair stand test versus worse or no improvement in the chair stand test as the dependent variable. In all models, one explanatory variable was group assignment (control or intervention). The estimate was further adjusted for age (dichotomised according to ⩾70 and <70 years), gender and comorbidity. Charlson Comorbidity Index was dichotomied according to no comorbidity versus any comorbidity.

Statistical significance was set as a *P* value < 0.05. The statistical Package for the Social Sciences (SPSS), version 25, was used for the analyses (SPSS Inc., Chicago, IL, U.S.A.).

## Results

**Figure 1.**
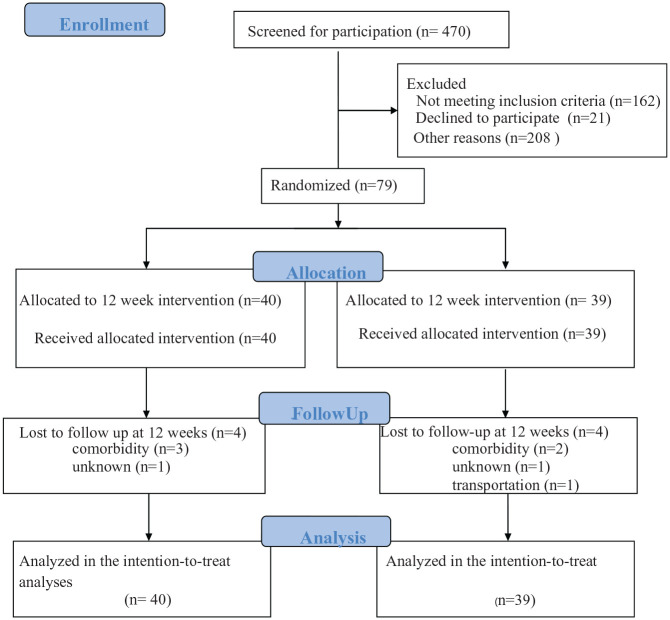
Flow chart of the study participants through the phases of the randomised controlled trial. Data were collected at Uppsala University Hospital, Sweden between November 1st, 2016 and December 18th, 2018. Individuals were randomised within one week after baseline assessment. No adverse events were reported.

Of 470 patients assessed for eligibility, 391 were excluded. Figure 1 shows a CONSORT flow diagram of the inclusion and retention process. At three months, 71 individuals (90%) remained in the study, including 36 in the intervention group (Figure 1). Thirteen individuals (five from the intervention and eight from the control group, respectively) received immediate rehabilitation for more than one occasion after discharge from the stroke unit.

Table 1 shows that at baseline 57 (72%) had suffered from an ischemic stroke, 9 (11%) intracerebral haemorrhage and 13 (16%) transient ischemic attack. Mean (SD) age was 63.9 (10.4) years and 29 (37%) were women. The two treatment groups were balanced (Table 1) at baseline; for example, six and seven of the patients with transient ischemic attack were randomised to the control and intervention group, respectively. Fifty-three of the participants were married, and 40 individuals reported a higher level of education.

**Table 1.** Patient characteristics at baseline.

	Text messaging (*n* = 40)	Control group (*n* = 39)
Age (years), mean (SD)	63.9 (10.1)	63.9 (10.8)
Sex, female, *n* (%)	16 (40.0)	13 (33.3)
Body Mass Index, mean (SD)	27.3 (4.2)	27.7 (4.9)
Montreal Cognitive Assessment scale, score, mean (SD)	26.3 (2.2)	25.7 (2.8)
Cerebral infarction *n* (%)	29 (72.5)	28 (71.8)
Intracerebral haemorrhage, *n* (%)	4 (10.0)	5 (12.8)
Transient ischemic attack, *n* (%)	7 (17.5)	6 (15.4)
Thrombolysis, *n* (%)	2 (5.0)	2 (5.1)

The baseline assessment was performed with a mean (SD) of 6 (4.4) days after the acute event. Online supplementary data show information on blood pressure, cardio-metabolic risk factors, stress profile, details of the comorbidity index, the physical activity scale and the modified Rankin Scale.

Adherence to the recommended interventions ranged from 60%–100% with a median value of 86%, according to the training diaries. Three individuals in the intervention group did not return the training diary but provided step counts. The sensitivity analyses showed overall similar results in the complete case analyses as in the intention to treat analyses. Thus, only intention-to-treat analyses are presented.

### Effects on walking performance and five times sit to stand test (primary outcomes)

The intervention group showed a significant improvement in the six-minute walking test at the three-month follow-up compared to the control group, that is, a median difference (IQR) of 48 (90) metres compared to 19 (66) metres; effect size, 0.64; *P* = 0.037 (Table 2). Moreover, the intervention group displayed improvement in the chair stand test after three months compared to the control group, that is, a median difference (IQR) of −1.4 (3.2) seconds compared to −0.7 (2.2) seconds; effect size, 0.64; *P* = 0.034 (Table 2).

**Table 2.** Baseline and follow-up outcome measures by group, intention-to-treat analysis. Data are presented as medians (IQR).

Characteristics	**Intervention group (*n* = 40)**		**Control** group **(*n* = 39)**		**Between** group differences		
	Baseline	Three-month follow-up	Baseline	Three -month follow-up	Median estimate (95% CI)	Effect size	*P-*value
**Six-minute Walk Test, m**	476 (138)	548 (142)	497 (146)	520 (173)	30 (55 to 1)	0.64	0.037
**Chair-stand Test, seconds**	11.6 (2.2)	9.6 (1.9)	11.4 (2.8)	10.5 (2.4)	−0.88 (–0.02 to −1.72)	0.64	0.034
**10-metre Walk Test, m/s**	1.22 (0.33)	1.31 (0.36)	1.27 (0.38)	1.31 (0.41)	0.01 (−0.05 to 0.07)		0.56
**Short Physical Performance Battery, score (0–12)**	11 (1)	12 (0)	11 (2)	12 (1)	0 (0 to 0)		0.86

CI indicates confidence interval. Significance of Between Group Differences was analysed bythe Mann-Whitney U test. The median estimate was calculated using the Hodges-Lehmann test. The significance level was set at *P* < 0.05.

Supplemental Table S1 displays the multivariate logistic regression analyses, which indicates that the text-message guided training was independently associated with a greater improvement in the six-minute walk test even after adjustment for age, gender and comorbidity.

Similar multivariate logistic regression analysis also shows that the text message guided training was independently associated with an improvement in the Chair Stand Test after adjusting for age, gender and comorbidity (Supplemental Table S2).

### Effects on physical function (secondary outcomes)

The 10-metre walk test and the short physical performance battery did not show any significant between-group changes at follow-up (Table 2).

Pedometer data provided from 33 of the 40 individuals in the intervention group showed a mean increase in the number of steps, from the first to the last week of the intervention, from around 6500 to 8000 steps (95% CI: 712 to 2380), *P* = 0.001, corresponding to a 23% increase. There was no monitoring of the control participants’ steps or physical activities during the study period.

No adverse events, for example, cardiovascular or cerebrovascular events, fall-related fractures, or syncope requiring hospitalisation were reported during the three months of the intervention.

## Discussion

Receiving mobile phone text messages with training instructions, combined with keeping a daily training diary and using a pedometer in the beginning and end of the intervention period, was superior to current standard care (absent of specific exercise recommendations) regarding walking performance and lower body strength in patients soon after stroke or with a transient ischemic attack. Many factors beyond motor impairment and physical function may explain the variation in overall mobility function after a stroke event.^1,7,29^ However, the randomised design of the study distributes such post-stroke conditions; for example, cardiac diseases, depression and fatigue, that impact mobility equally between the groups.

The focus of the study was to evaluate a telehealth intervention in order to facilitate adherence to the physical activity guidelines for individuals after stroke and transient ischemic attack. The intention was to include low- to moderate intensity aerobic activity, as well as muscle strengthening exercises, in order to achieve at least 30 minutes of daily exercise in the intervention group after three months.^10,30^ Although the degree of brain injury differs, individuals with stroke and transient ischemic attack share risk-factor profile and have a similar risk of recurrent disease. Therefore, it is important to pay attention to both conditions in risk-factor management, including physical activity and sedentary behaviour.

Cognition is often affected after a stroke event. At study start, the absence of cognitive impairment was a mandatory inclusion criterion. Because the recruitment rate was slow in the beginning of the study an amendment to the protocol was introduced; that is, to accept patients with Montreal Cognitive Assessment <23 points. The condition for this protocol change was that there was a spouse available to assist with the training instructions. Thus, 45% of the participants displayed some cognitive impairment at baseline. Cognitive impairments in close proximity to a stroke are often reversible. Unfortunately, cognitive function was not assessed at follow-up.

Studies on telehealth interventions appear scarce. The authors are unaware of and could not find any literature using telehealth strategies and messaging to increase walking performance in individuals soon after stroke or transient ischemic attack. However, studies on group training in similar patient groups but without telehealth support are available. For example, the results of the present study are in line with those of a randomised controlled trial with intensive aerobic exercises twice weekly to 56 discharged patients following a stroke.^31^ Two sets of eight-minute ergometer cycling were combined with flexibility exercises. The control group did not receive any organised training sessions. The six-minute as well as the 10-metre walking tests, health-related quality of life and balance were significantly improved by the intensive aerobic exercise. The major design difference between the two studies is that the training in the reviewed study was group based, whereas the present study was designed to encourage home-based individual training through the use of a mobile phone.

In addition to walking performance, lower body strength was assigned to be a primary outcome measure in the present study. It was encouraging to observe improvements in the chair-stand test after three months with the short message service-guided training instructions. Our findings are in agreement with a Cochrane report,^32^ including 603 participants after a stroke, which concludes that there is moderate evidence that interventions to improve sit-to-stand have a beneficial effect. The chair stand exercises in our study may have improved balance for some of the study participants, which might have contributed to the observed improvement.

It is likely that the good compliance with the training was one reason for the improvement in overall mobility; that is, the six-minute walk test, increased number of steps per week and lower body strength, in the short message service intervention group. Indeed, compliance to the short message serviceguided training was above 60%. The possibility to exchange walking for other activities, for example, outdoor cycling, aerobic classes or heavy gardening may have further increased compliance to the programme.

There were no improvements in the secondary outcomes and composite measures of mobility, that is, the 10-metre walk test or the short physical performance battery, by the intervention in this study. The short physical performance battery is an ordinal scale ranging from 0 to 12 points. Already at the study start the participants scored an average of 11 points. Thus, improvements would be difficult to achieve in this particular outcome variable; that is, a so-called ceiling effect was observed. Neither the 10-metre walk appeared to be sensitive enough to detect differences. Perhaps, measures of balance, like the Berg balance scale^33^ or the mini-BESTest^34^ had been more responsive for this population with minimal disability.

Wearable sensor technologies and telehealth programmes as a complement to conventional therapy in individuals with stroke are under development and forthcoming research is ongoing.^35,36^ It could be speculated that exercise instructions delivered via telehealth might provide greater intensity of therapy without additional physiotherapist time, and thus making home-based stroke rehabilitation more accessible. Moreover, the training instructions via mobile-phone delivered text messages mighthelp to overcome barriers to start exercise, and to serve as remindersfor exercise.

To fully evaluate the potentially beneficial effects of the telehealth approach, it would be necessary to have another control group that practised physical activity by guidance from physiotherapists at a rehabilitation service. This indicates that the study has limitations that need to be acknowledged. Another is that we had to screen a substantial number of individuals to reach our target sample size, which could have introduced a selection bias. All our participants were discharged from the hospital to independent living. In Sweden, about 77% of stroke patients are discharged to their regular home, although they have not all had a mild stroke, and being unable to walk at the time of discharge.^37^ We cannot say that this model of text-messaging will also work for individuals with more limited mobility after stroke that return home. Individuals with known severe psychiatric problems or with visual impairments; for example, visual field losses might have a greater need for physiotherapist-led training. Furthermore, other post-stroke patients may suffer from aphasia or cognitive problems, with ensuing difficulties to use a mobile phone or a training diary. For these reasons, the feasible population of patients with stroke or transient ischemic attack for this kind of intervention may be restricted. Another limitation is that the study design did not allow the examiner to be blinded to the intervention at the follow-up examination. The participants were allowed to communicate with the examiner, and although not encouraged to reveal their group allocation, about one in four did so. Thus, it cannot be excluded that the non-blinded follow-up assessment may have biased the outcome. Still, the objective nature of many of the outcome measurements, for example, walking performance, give some assurance that the results are robust.

Measuring steps only the first and last week of intervention is a potential limitation. However, when the study was planned this was considered sufficient to evaluate progression in walking distance. Positive features of the pedometer could be that it gave the users direct feedback on their walking performance. This may have affected the motivation to walk, as well as to follow the sms-delivered training instructions. Measures of steps are easy to understand and can easily be translated to clinical practice, wellness programmes and public health recommendations. Mobile phone applications that counts steps (pedometers) is readily used in clinical rehabilitation for registration of walking performance. For some individuals it was difficult to fill in the training diary, which may make such diaries less useful. Furthermore, in a pilot study^11^ we intended to compare exercise instructions by text messages with recorded videos with verbal exercise instructions given by a physiotherapist and sent as links to the individuals. However, receiving text messages was clearly preferred.^11^ The text messaging method might be used for other stroke home-based rehabilitation activities, for example, stretching a hypertonic arm or training of other lost skills.

The major strength of this clinical study is its prospective and randomised controlled design, with a predefined intervention that could be performed almost anywhere. Furthermore, we used objective measurements with acceptable psychometric properties, commonly used in stroke research. Both men and women were included, and the age range was broad. The study was well-powered and had a lower drop-out rate than assumed in our power-calculation, that is, only 10% dropouts.

Improvements in physical functioningis worthwhile for enhancing ADL in patients that have suffered a stroke or a transient ischemic attack. Likewise, the risk factor burden related to aberrations in glucose metabolism, dyslipidemia and hypertension could be expected to decrease by increased physical activity.The use of mobile phones is today a common way to communicate (even among older people). The described exercises can be performed in almost any environment, and the method of delivering the instructions is cost-effective since no personnel is needed.

In summary, the STROKEWALK Study indicates that daily mobile phone delivered instructionalmessages (short message service) combined with training diaries and pedometers result in better walking performance and chair-stand ability compared with current standard care in post-stroke community-dwelling patients. Further studies are needed to confirm these assumptions. Research is also needed to evaluate this type of intervention in more disabled people, as well as in patients with cognitive deficits.

**Clinical messages**

- Three months of daily mobile phone exercise instructional text messages led to greater improvement in walking performance and lower body strength when compared with currentpractice of absent routine recommendations of exercise.
- The text-messages were supported by daily training diaries and a pedometer, in the beginning and end of the study period.

## Supplemental Material

Table_S1 – Supplemental material for Effects on walking performance and lower body strength by short message service guided training after stroke or transient ischemic attack (The STROKEWALK Study): a randomized controlled trial Click here for additional data file. Supplemental material, Table_S1 for Effects on walking performance and lower body strength by short message service guided training after stroke or transient ischemic attack (The STROKEWALK Study): a randomized controlled trial by Birgit Vahlberg, Erik Lundström, Staffan Eriksson, Ulf Holmbäck and Tommy Cederholm in Clinical Rehabilitation

## Appendix

**Appendix 1.** Examples of the mobile phone delivered text-messages in Swedish and English.

**Vecka 1:** Idag skall Du promenera 15 minuter. Borg 12: (lätt).Gör 10 uppresningar från sittande, utan stöd. Upprepa 3 gånger. **Week 1**: ‘Today, you shall walk for 15 minutes, Borg scale 12 (light). Stand up from sitting 10 times, without support, repeat 3 times’.
**Vecka 3:** Idag skall Du promenera 15 minuter. Borg 13: (något ansträngande).Gör 10 uppresningar från sittande, utan stöd. Upprepa 3 gånger. **Week 3:** ‘Today, you shall walk for 15 minutes, Borg scale 13 (somewhat strenuous). Stand up from sitting 10 times, without support, repeat 3 times’.
**Vecka 8:** Idag skall Du promenera 25 minuter. Borg 14: (något ansträngande).Gör 15 uppresningar från sittande, utan stöd. Upprepa 3 gånger. **Week 8:** ‘Today, you shall walk for 25 minutes, Borg scale 14 (somewhat strenuous). Stand up from sitting 15 times, without support, repeat 3 times’.
**Vecka 9-12:** Intervaller: (måndag, torsdag och lördag) Gå snabbt 4 minuter, Borg 15 (ansträngande) Upprepa 4 gånger. Gör 15 uppresningar från sittande, utan stöd. Upprepa 3 gånger. Vila: Onsdagar. Promenader: (tisdag, fredag och söndag). Idag skall Du promenera 30 minuter, Borg 14:( något ansträngande). Gör 15 uppresningar från sittande, utan stöd. Upprepa 3 gånger. **Week 9-12:** Intervals: (Monday, Thursday and Saturday). Walk fast for 4 minutes, Borg 15 (strenuous) Walk slower for 3 minutes, Borg 12 (light) Stand up from sitting 15 times, without support, repeat three times’. Resting: Wednesdays. Regular walks (Tuesday, Friday and Sunday). Today, you shall walk for 30 minutes, Borg scale 14 (somewhat strenuous). ‘Today, you shall walk for 30 minutes, Borg scale 14 (somewhat strenuous). Stand up from sitting 15 times, without support, repeat three times’.
