# Supplementary material for: Effects on walking performance and lower body strength by short message service guided training after stroke or transient ischemic attack (The STROKEWALK Study): a randomized controlled trial
Source: Clin Rehabil. 2020 Sep 18;35(2):276–87. doi: 10.1177/0269215520954346 (PMC7874373; doi:10.1177/0269215520954346)
Supplement: Table_S1 – Supplemental material for Effects on walking performance and lower body strength by short message service guided training after stroke or transient ischemic attack (The STROKEWALK Study): a randomized controlled trial [file Table_S1.pdf]

Table S1. Logistic regression showing Odds Ratios 95% Confidence Interval (CI)) for improvement; i.e. odds for belonging to the group with a change in 6-minute Walk Test of more than the median improvement of 32 meters (=1) vs. improvement of less than 32 meters (=0). Data from 79 cases after stroke or transient ischemic attack.

	Univariate model			Multivariate model		
	Odds ratio	95%CI	*P*	Odds ratio	95%CI	*P*
Explanatory variables						
**Group**						
Control (reference)						
SMS	4.67	1.81 to 12.05	0.001	5.77	2.00 to 16.58	0.001
**Age,** years						
≥ 70 (reference)						
< 70	3.93	1.40 to 11.07	0.010	4.38	1.60 to 16.08	0.006
**Gender**						
Male (reference)						
Female	0.80	0.32 to 1.99	0.62	0.63	0.22 to 1.82	0.40
**CCI,** index						
0 (reference)						
1-6	0.58	0.23 to 1.43	0.23	0.59	0.21 to 1.66	0.32

Abbreviations: CCI; Charlson Comorbidity Index. The model is adjusted for age at baseline, gender, and comorbidity.

Chi-square (χ^2) = 3.30; P = 0.86 (Hosmer-Lemeshow Test); Nagelkerke R square = 0.31

Table S2. Logistic regression providing odds for improvement in Chair-stand Test dichotomized into "unchanged or worse" = 0 and "improved"= 1, in 79 cases after acute stroke or transient ischemic attack.

	Univariate model			Multivariate model		
	Odds ratio	95%CI	*P*	Odds ratio	95%CI	*P*
Explanatory variables						
**Group**						
Control (reference)						
SMS	2.78	1.02 to 7.59	0.046	2.90	1.01 to 8.31	0.047
**Age,** years						
≥70 (reference)						
<70	2.09	0.76 to 5.76	0.15	2.26	0.78 to 6.57	0.13
**Gender**						
Male (reference)						
Female	0.95	0.35 to 2.57	0.92	0.86	0.30 to 2.49	0.78
**CCI,** index						
0 (reference)						
1-6	0.45	0.17 to 1.19	0.11	0.45	0.16 to 1.24	0.12

Abbreviations: CCI; Charlson's Comorbidity Index. The model is adjusted for age at baseline, gender, and comorbidity.

Chi-square (χ^2) = 7.315; $P = 0.40$ (Hosmer-Lemeshow Test); Nagelkerke R square = 0.15

Appendix: Examples of the mobile phone delivered text-messages in Swedish and English.
**Vecka 1:** Idag skall Du promenera 15 minuter. Borg 12: (lätt).<br>Gör 10 uppresningar från sittande, utan stöd. Upprepa 3 gånger.<br><br>**Week 1**: "Today, you shall walk for 15 minutes, Borg scale 12 (light). Stand up from sitting 10 times, without support, repeat 3 times."
**Vecka 3:** Idag skall Du promenera 15 minuter. Borg 13: (något ansträngande).<br>Gör 10 uppresningar från sittande, utan stöd. Upprepa 3 gånger.<br><br>**Week 3:** "Today, you shall walk for 15 minutes, Borg scale 13 (somewhat strenuous). Stand up from sitting 10 times, without support, repeat 3 times."
**Vecka 8**: Idag skall Du promenera 25 minuter. Borg 14: (något ansträngande).<br>Gör 15 uppresningar från sittande, utan stöd. Upprepa 3 gånger.<br><br>**Week 8:** "Today, you shall walk for 25 minutes, Borg scale 14 (somewhat strenuous). Stand up from sitting 15 times, without support, repeat 3 times."

Vecka 9-12: Intervaller: (måndag, torsdag och lördag)

Gå snabbt 4 minuter, Borg 15 (ansträngande)

Gå långsammare 3 minuter, Borg 12 (Lätt)

Upprepa 4 gånger.

Gör 15 uppresningar från sittande, utan stöd. Upprepa 3 gånger.

Vila: Onsdagar.

Promenader: (tisdag, fredag och söndag). Idag skall Du promenera 30 minuter, Borg 14:(något ansträngande).

Gör 15 uppresningar från sittande, utan stöd. Upprepa 3 gånger.

Week 9-12: Intervals: (Monday, Thursday and Saturday).

Walk fast for 4 minutes, Borg 15 (strenuous)

Walk slower for 3 minutes, Borg 12 (light)

Stand up from sitting 15 times, without support, repeat three times.”

Resting: Wednesdays.<br><br>Regular walks (Tuesday, Friday and Sunday). Today, you shall walk for 30 minutes, Borg scale 14 (somewhat strenuous).<br><br>“Today, you shall walk for 30 minutes, Borg scale 14 (somewhat strenuous). Stand up from sitting 15 times, without support, repeat three times.”

Supplementary data. Baseline characteristics of the study participants

	**Exercise SMS (n = 40)**	**Control Group (n = 39)**
Systolic blood pressure (mmHg), mean (SD)	129.1 (14.5)	132.9 (18.8)
Diastolic blood pressure (mmHg), mean (SD)	75.1 (11.4)	77.1 (10.6)
Cardio-metabolic risk factors, n (%)		
Systolic blood pressure ≥ 140 (mmHg)	10 (25)	17 (43.6)
Diastolic blood pressure ≥ 90 (mmHg)	7 (17.5)	6 (15.4)
Smoking	5 (13.2)	3 (7.5)
Diabetes	5 (12.5)	7 (17.9)
Hypertension	21 (52.5)	24 (61.5)
Hypercholesterolemia	6 (15.0)	11 (28.2)

Stress profile, score (0-60), mean (SD)	19.9 (9.6)	21.0 (12.7)
Stress profile, score, n (%)		
<25	27 (67.5)	25 (64.1)
25-34	11 (27.5)	7 (17.9)
≥35	2 (5.0)	7 (17.9)
Modified Rankin Scale at inclusion, n (%)		
0	7 (17.5)	4 (11.1)
1	25 (62.5)	28 (71.8)
2	8 (20.0)	7 (17.9)
Saltin Grimby Physical Assessment Level Scale, level		
1 (sedentary)	2 (5.0)	9 (23.0)
2 (some light physical activity)	29 (72.5)	24 (61.5)
3 (regular moderate physical activity)	9 (22.5)	6 (15.4)

4 (hard physical training)	0	0
Charlson Comorbidity Index, n (%)		
0 (no comorbidity)	25 (62.5)	22 (55.0)
1		4 (10.3)
2	9 (22.5)	6 (15.4)
3	3 (7.5)	2 (5.1)
4	2 (5.0)	3 (7.7)
5		2 (5.1)
6	1 (2.5)	

Blood pressure values are the last registration before discharge from the hospital.

Charlson Comorbidity Index is calculated from the data in the medical journals upon discharge from the hospital.
